# Depression and Anxiety Scores Are Associated with Amygdala Volume in Cushing's Syndrome: Preliminary Study

**DOI:** 10.1155/2017/2061935

**Published:** 2017-05-18

**Authors:** Alicia Santos, Esther Granell, Beatriz Gómez-Ansón, Iris Crespo, Patricia Pires, Yolanda Vives-Gilabert, Elena Valassi, Susan M. Webb, Eugenia Resmini

**Affiliations:** ^1^Endocrinology/Medicine Departments, Hospital Sant Pau, Centro de Investigación Biomédica en Red de Enfermedades Raras (CIBER-ER, Unidad 747), IIB-Sant Pau, ISCIII and Universitat Autònoma de Barcelona (UAB), Barcelona, Spain; ^2^Neuroradiology Unit, Hospital de Sant Pau and IIB-Sant Pau, UAB, Barcelona, Spain; ^3^INNDACYT, Avda. Europa 20, Planta Baja Puerta D 08907, Hospitalet de Llobregat, Spain

## Abstract

**Introduction:**

Cushing's syndrome (CS) has repeatedly been associated with hippocampal volume reductions, while little information is available on the amygdala, another structure rich in glucocorticoid receptors. The aim of the study was to analyze amygdala volume in patients with CS and its relationship with anxiety, depression, and hormone levels.

**Material and Methods:**

39 CS patients (16 active and 23 patients in remission) and 39 healthy controls matched for age, sex, and education level completed anxiety (STAI) and depression tests (BDI-II) and underwent a 3 Tesla brain MRI and endocrine testing. Amygdala volumes were analysed with FreeSurfer software.

**Results:**

Active CS patients had smaller right (but not left) amygdala volumes when compared to controls (*P* = 0.045). Left amygdala volumes negatively correlated with depression scores (*r* = −0.692, *P* = 0.003) and current anxiety state scores (*r* = −0.617, *P* = 0.011) in active CS patients and with anxiety trait scores (*r* = −0.440, *P* = 0.036) in patients in remission. No correlations were found between current ACTH, urinary free cortisol or blood cortisol levels, and amygdala volumes in either patient group.

**Conclusion:**

Patients with active CS have a smaller right amygdala volume in comparison to controls, while left amygdala volumes are associated with mood state in both patient groups.

## 1. Introduction

Cushing's syndrome (CS) is a rare disease due a cortisol excess. Its symptoms include facial plethora, muscle weakness, skin symptoms (red or purple striae and easy bruising), bone loss, cardiovascular risk factors (hypertension, diabetes and impaired glucose tolerance, dyslipidemia, coagulopathy, and central obesity), fatigue, lack of libido, emotional lability, depression, anxiety, and cognitive alterations [1–5]. Even after cure, patients can still have physical and psychological comorbidities, which may negatively impact on quality of life [6, 7].

Cortisol excess has been associated with structural alterations of the brain. Studies in active disease have found smaller whole brain, hippocampal, and cerebellar volumes in comparison to controls, while there is still some controversy on possible recovery after cure [8–11]. Interestingly, less attention has been devoted to the amygdala, a brain structure with an important role in emotional response that is also a target area for glucocorticoid hormones through the activation of glucocorticoid and mineralocorticod receptors [12–15].

The few studies in CS focusing on the amygdala have reported interesting data, part of them in children and adolescents. Smaller amygdala volumes have been found in children with CS when compared to controls, manually tracing the amygdalar structure on MRI. In contrast to total cerebral volume, this volumetric reduction did not seem to reverse after cure [16]. In adult patients in remission, no differences in volumes were found for amygdala using voxel based morphometry compared to controls [13]. Regarding functional studies, in adolescents, greater left amygdala activation was found during face encoding tasks [17], while in adult patients in remission no differences in amygdalar activation were found when processing emotional faces [18]. To our knowledge, no data are available on amygdalar volume in adult patients with active CS.

The aim of this study was to analyze amygdala volume in patients with CS (both active and in remission) and relate it to anxiety, depression, hormonal levels, and both duration of hypercortisolism and delay to diagnosis.

## 2. Material and Methods

### 2.1. Patients

The sample included 39 patients with CS (16 patients with active hypercortisolism and 23 patients in remission) and 39 healthy controls, which were matched for sex, age (±3 years), and years of education (±3 years). Patients were addressed and recruited during their routine follow-ups in the hospital. Controls were recruited from other studies and from the blood donor's center of the hospital. Before enrolment, both patients and controls signed an informed consent. The study was approved by the ethics committee of the hospital.

Exclusion criteria for patients with CS included the following: age above 65 years, known prior cerebrovascular disease, severe neurological or psychiatric illness, growth hormone (GH) deficiency, and history of drug or alcohol abuse. For controls, the same criteria were considered, and endocrine disease and glucocorticoid exposure were additional exclusion criteria. As usual in MRI studies, only right handed subjects (CS patients and controls) were included (Edinburgh Handedness Inventory > 80) [19].

Patients were divided into two categories according to disease activity. They were considered in remission after surgery if morning cortisol suppression (<50 nmol/l) was observed after 1 mg dexamethasone overnight [20]; if adrenal insufficiency was demonstrated and if repeated 24 h urinary free cortisol measures were within normal parameters (<280 nmol/24 h). Patients who did not fulfil these criteria were considered to have active CS. All patients considered in remission met the criteria for remission for at least 6 months. None of the active patients had undergone surgery to treat CS, except one who recurred after initial remission of CS.

### 2.2. Biochemistry, Clinical Information, and Questionnaires

Participants underwent testing for 24-hour urinary free cortisol (determined with a commercial radioimmunoassay, Coat-A-Count Cortisol, and Siemens), Plasma ACTH was measured by chemiluminiscent immunometric assay (Immulite 2000, Siemens Healthcare Diagnostics Products Ltd., Llanberis, UK). Serum cortisol was measured by electrochemiluminescent immunoassay (Modular Analytics E170, Roche Diagnostics GmbH, Mannheim, Germany).

Clinical information was obtained from the patients' clinical files. The estimation of duration of hypercortisolism was calculated as the months from symptom onset to the date of remission of hypercortisolism (or current date for active patients, where remission had not been achieved). Delay to diagnosis was considered as the months elapsed from symptom onset to the final diagnosis of CS.

Participants also completed two questionnaires to assess depression and anxiety. Beck Depression Inventory II (BDI-II) is a self-reported questionnaire to assess the severity of depressive symptoms. It consists of 21 items (scored from 0 to 3), taking about five minutes to complete. The total score can range from 0 to 63, and higher scores indicate higher severity of depressive symptoms. Scores can be interpreted as minimal depression (0–13), mild depression (14–19), moderate depression (20–28), and severe depression (29–63) [21]. State Trait Anxiety Inventory (STAI) is also a self-reported questionnaire with two subscales that evaluate state anxiety (related to the present moment) and trait anxiety (a more general personal characteristic). Both subscales consist of 20 items (scored from 0 to 3). The total scores for both state and trait subscales can range from 0 to 60. For result interpretation, total scores are transformed into percentiles or decatypes. In this study we decided to use percentiles. Higher scores for both total scores and percentiles correspond to higher anxiety levels [22].

### 2.3. Magnetic Resonance Imaging (MRI)

All participants underwent 3 Tesla magnetic resonance imaging (MRI). Imaging was obtained using a Philips Achieva facility (software version 2.1.3.2) and a dedicated acquisition protocol (3DMPRAGE whole brain sequence; repetition time = 6.7 msec; echo time = 3.1 msec, 170 slices; voxel size = 0.889 × 0.889 × 1.2).

Images were postprocessed in the Port d'Informació Científica (PIC) of the Universitat Autònoma de Barcelona and volumes of the left and right amygdala were obtained. Specifically, volumetric segmentation was performed automatically using FreeSurfer version 5.3 image analysis software (http://surfer.nmr.mgh.harvard.edu/). It is composed of 170 HP blades with two quad-cores CPU (Hewlett Packard, Palo Alto, CA), each one with 16 GB of RAM, running over Scientific Linux version 5 (https://www.scientificlinux.org/). FreeSurfer's processing includes removal of nonbrain tissue using a hybrid watershed/surface deformation procedure [23], motion correction, automated Talairach transformation, and segmentation of the subcortical white matter and deep grey matter volumetric structures [24, 25]. The PICNIC tool (https://neuroweb.pic.es) was used for postprocessing. Both the visual check and the automated image processing were performed by a single blinded investigator.

All volumetric scores were normalized to the estimated intracranial volume of each individual, as previously described [11].

### 2.4. Statistics

Statistical analysis was performed using IBM SPSS 22 software (SPSS Inc., Chicago, IL, USA). The Kolmogorov-Smirnov test was used to assess normal distribution. For comparisons of amygdalar volumes between the three groups (active patients, patients in remission, and controls), ANOVA followed by a posthoc analysis (Bonferroni) was used. A three-way ANOVA was used to rule out the possible effect of glucocorticoid replacement, antidepressants, or radiotherapy on our results.

For nonnormal data comparisons (specifically for BDI-II and STAI) the Kruskal-Wallis test was used, and the posthoc analysis was performed with a Mann–Whitney *U*-test. Correlations among variables were assessed using Sperman's rho. For correlations including biochemical parameters (urinary free cortisol, blood cortisol, and ACTH), patients taking hydrocortisone were excluded from the analysis. Statistical differences were considered significant when *P* < 0.05.

## 3. Results

As expected from previous matching, no differences were found for sex, age, or education level between the groups.  Table 1 summarizes their clinical and demographic characteristics.

Active patients had a smaller right (but not left) amygdala volume when compared to controls (*P* = 0.045). No differences were found for amygdala volumes when comparing patients in remission and controls. Mean amygdala volumes can be found in Table 2. Results did not change when patients taking antidepressives (1 in the active group, who was active due to a recurrence of CS, and 5 in the group of patients in remission) were excluded, together with their matched controls, although a marginal tendency seemed to show up for left amygdala volumes when comparing the active and the control group (right *P* = 0.035; left *P* = 0.084). Results did not change when the patient on long-term medical treatment for hypercortisolism and her matched control were excluded from the analysis (right amygdala *P* = 0.033; left *P* = NS).

In order to rule out the possible effect of glucocorticoid replacement, antidepressants, or radiotherapy on our results, a three-way ANOVA including these three factors was performed. The analysis revealed that none was significant for amygdala volumes.

**Figure 1:**
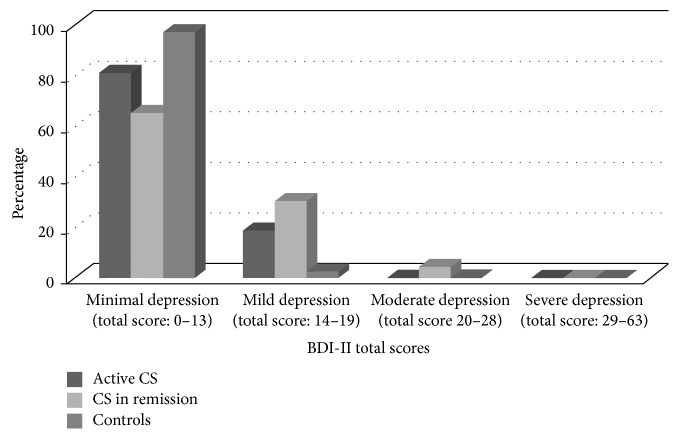
Details on the percentage of patients and controls presenting different depression scores (Beck Depression Inventory II).

**Figure 2:**
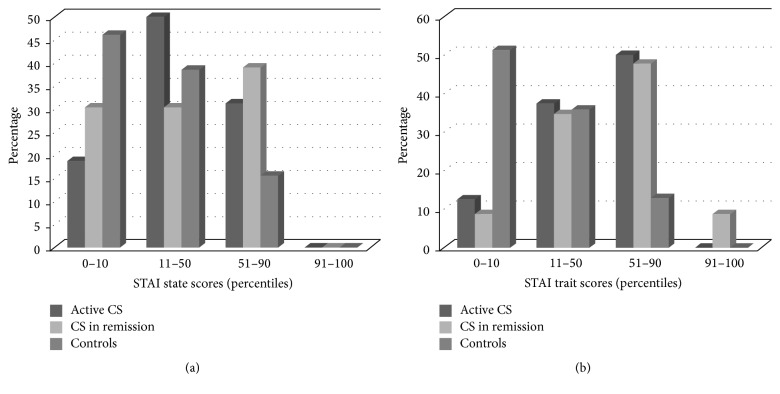
Details on anxiety scores (State Trait Anxiety Inventory) expressed in percentiles for patients and controls (higher percentiles represent higher anxiety levels). STAI manual does not provide a range interpretation, so for descriptive reasons percentile scores have been divided in four groups of percentiles. Higher scores indicate higher anxiety.

Regarding neuropsychological findings, both patient groups had higher depression and anxiety scores than controls (BDI-II: active *P* < 0.001, in remission *P* < 0.001; STAI state state: active *P* = 0.005, in remission *P* = 0.019; STAI trait: active *P* = 0.001, in remission *P* < 0.001). Descriptive details on the degree of severity of anxiety and depression can be found in Figures 1, 2(a), and 2(b).

Correlations between amygdala volumes and depression and anxiety scores were analysed. In active CS patients, left (but not right) amygdala volume negatively correlated with depression scores (*r* = −0.692, *P* = 0.003) and current anxiety state scores (*r* = −0.617, *P* = 0.011). In patients in remission, left (but not right) amygdala volume negatively correlated with anxiety trait scores (*r* = −0.440, *P* = 0.036). No correlations were found between amygdala volumes and depression and anxiety scores in controls. Mean duration of hypercortisolism and delay to diagnosis did not correlate with amygdala volumes in any of the patient groups (*P* = NS).

A final analysis was devoted to the correlation between amygdala volumes and biochemical results. Neither ACTH, urinary free cortisol, nor blood cortisol levels correlated with amygdala volumes in active patients or patients in remission. In contrast, right amygdala volumes negatively correlated with ACTH (*r* = −0.344, *P* = 0.05), 24 hour-urinary free cortisol (*r* = −0.354, *P* = 0.047), and blood cortisol (*r* = −0.420, *P* = 0.009) in controls.

## 4. Discussion

These results show that active CS patients had smaller right amygdala volumes than controls. This suggests that glucocorticoid excess due to CS may lead to amygdala volume shrinkage, as previously reported for other brain structures, like the hippocampus and the cerebellum [8–10]. The mechanisms related to this shrinkage are probably multifactorial and could include cell death (apoptosis), reduced neurogenesis, or dendritic structure modification, among others [26–31].

The fact that smaller amygdala volumes were not found in patients in remission leads us to hypothesize that some sort of volumetric increase may occur after biochemical cure. In fact, for the hippocampus a 10% increase has been demonstrated after disease remission [32]. Similar results have been found for the cerebellum, where smaller grey matter volumes were found in active patients compared to controls but did not differ when comparing with patients in remission [9, 13, 33].

It was initially surprising to find that only the right amygdala (but not the left) was smaller in the active group compared to controls. However, it important to highlight that the two amygdala structures are not equal and may have different roles in emotional processing [34]. Interestingly, we found one study in unmedicated patients with major depression who also had smaller right amygdala volumes in comparison to controls, while a marginal nonsignificant tendency was found for left amygdala volumes [35]. Similarly, patients with treatment-resistant depression and elderly depressed patients also have smaller right (but not left) amygdala volumes in comparison to controls [36, 37]. Therefore, mood state could have played a role in our findings. However, due to the small sample size, we can not rule out that volumetric differences may also occur in the left amygdala volumes.

The fact that both active CS patients and those in remission had higher anxiety and depression levels than controls is in line with previous findings, where both psychological comorbidities have been described [4, 13, 38–40]. It is important to highlight that most of the patient's scores were not extremely high.

For depression, mild depression scores were present in 18.8% of active patients and 30.4% of patients in remission, while moderate depression scores were not present in the group of active patients and only in 4.3% of patients in remission. This was surprising since 50 to 80% of the patients with active disease met criteria for major depression [1]. A possible explanation may be that most of the active CS patients were on medical treatment to reduce their cortisol levels (which has been reported to have a positive effect on mood alterations) [41, 42], and one patient was taking antidepressives. For patients in remission, five were also on antidepressants, which could have prevented impairment in mood state. Furthermore, GH deficiency, known to be associated with depression and anxiety, was an exclusion criterion in our study, but not in others, related to high depression levels [43].

Regarding anxiety, no active patients or controls were in the 91–100 percentile range (indicating the higher range of the normal curve and therefore higher anxiety [44]) for STAI state or trait, and only 8.7% of the patients in remission were in this range for STAI trait. Therefore, even if mean scores were higher than controls, most of the patient's scores were included in the normal range. As for depression, cortisol lowering medication may have improved previous higher anxiety status in active patients, and excluding patients with GH deficiency may have limited further finding [45].

Negative correlations were found between left amygdala volumes and depression and anxiety levels. These findings are in line with other populations, where left amygdala volumes have been negatively correlated with both state and trait anxiety levels in normal population and with anxiety state in panic disorder [46, 47]. In fact, in emotion studies, a lateralization to the left in amygdala activation has been reported, particularly for negative emotions, which may play a role in our results [48]. Left amygdala volume has also been related to perceived social support in normal subjects, following a positive association [49].

A meta-analysis on the amygdala volume in major depressive disorder reports smaller amygdala volumes in patients than controls, in studies including unmedicated patients (while normal or higher volumes than controls have been found in medicated patients) [50]. Interestingly, patients with active CS (where smaller amygdala volumes were found) often do not respond properly to antidepressives until cortisol is normalized. Therefore, even if depression was present, medication may not be indicated until remission of hyercortisolism [41, 42, 51].

No correlations were found between hormone levels and amygdala volumes, while intriguingly this was observed in normal controls. It is possible that, in normal population, hyperactivation of the HPA axis directly affects the amygdala, leading to a right amygdalar volume reduction. However, in patients having suffered chronic hypercortisolism, other mechanisms (as mood alterations) may also have been influencing amygdala volumes. Alternatively, it is possible that correlations were not found, as prior chronic hypercortisolism (even before diagnosis) could have already altered the amygdala volume at an earlier stage, without correlations with current hormone levels.

Limitations of this study include the small sample size, difficult to avoid in rare disease studies. The heterogeneity of the sample, including patients with different etiologies or having performed different treatments, could be a further limitation. This again is difficult to avoid in a rare disease like CS. We tried to reduce it by strict exclusion criteria (including GH deficiency). Other limitations include not having performed a complementary functional MRI acquisition (including performance of emotional tasks) or evaluating androgens. Finally the cross-sectional design precluded drawing any conclusions on the longitudinal course of amygdala volumes. Future studies should include a follow-up of active patients to establish if the amygdala volume increases after disease remission and include a higher number of patients taking or not antidepressives, in order to compare both groups. Furthermore, studies including both structural and functional MRI at the same time could clarify further implications of the data we found.

## 5. Conclusions

In conclusion, patients with active CS (but not patients in remission) have a smaller right amygdala volume in comparison to controls, while left amygdala volumes are associated with mood state in both patient groups.

## Figures and Tables

**Table 1:** Clinical characteristics of patients with Cushing's syndrome (CS) and controls.

	CS (*n* = 39)		Controls (*n* = 39)
	Active (*n* = 16)	In remission (*n* = 23)	
Age	44.1 ± 9.0	42.9 ± 10.6	42.6 ± 10.3
Sex (female/male)	14/2	19/4	33/6
Years of education	13.9 ± 2.7	13.2 ± 3.3	13.9 ± 3.3
Origin of CS (pituitary/adrenal/ectopic/AIMAH^†^)	10/4/1/1	20/3/0/0	—
Cortisol lowering medication (Metyrapone/Ketoconazole/Cabergoline/Losartan)	4/8/1/1	—	—
Mean length of treatment with cortisol lowering medication (months)	4.4 ± 4.2^*∗*^	—	—
Hydrocortisone replacement	—	8	—
Antidepressive medication	1	5	—
Recurrencies	1	3	—
Radiotherapy	—	5	—
Mean duration of hypercortisolism (months)	62.2 ± 59.1	63.1 ± 34.6	—
Delay to diagnosis (months)	49.1 ± 42.7	44.4 ± 36.1	—
Mean time of biochemical cure (months)	—	66.0 ± 69.0	—
Urinary free cortisol (nmol/24 h)	350.4 ± 293.1	124.8 ± 49.2	145.5 ± 88.9

^*∗*^Including the whole sample of patients taking cortisol-lowering medication except one patient with an ectopic ACTH secretion who had been taking medication for 120 months.

^†^AIMAH: ACTH-independent macronodular adrenal hyperplasia.

**Table 2:** Mean amygdala volumes in patients and controls.

	Active CS (*n* = 16)	CS in remission (*n* = 23)	Controls (*n* = 39)
Right amygdala (mm^3^)	1975.6 ± 392.2^*∗*^	2141.1 ± 319.4	2208.5 ± 274.9
Left amygdala (mm^3^)	1757.8 ± 290.8	1884.3 ± 277.6	1901.5 ± 242.6

^*∗*^Differences between active CS patients and controls (*P* < 0.05).
